# Effect of Short Attritor-Milling of Magnesium Alloy Powder Prior to Spark Plasma Sintering

**DOI:** 10.3390/ma13183973

**Published:** 2020-09-08

**Authors:** Peter Minárik, Mária Zemková, Michal Knapek, Stanislav Šašek, Jan Dittrich, František Lukáč, Jiří Kozlík, Robert Král

**Affiliations:** 1 Faculty of Mathematics and Physics, Charles University, 121 16 Prague, Czech Republic; 1zemkova.maria1@gmail.com (M.Z.); michal.knapek@gmail.com (M.K.); sasekstanislav@seznam.cz (S.Š.); dittrich.jan.cz@gmail.com (J.D.); lukac@ipp.cas.cz (F.L.); jiri.kozlik@mff.cuni.cz (J.K.); rkral@met.mff.cuni.cz (R.K.); 2 Nuclear Physics Institute CAS, Řež 130, 25068 Řež, Czech Republic; 3 Institute of Plasma Physics CAS, Za Slovankou 3, 182 00 Prague, Czech Republic

**Keywords:** magnesium, powder, spark plasma sintering, milling, mechanical properties, microstructure

## Abstract

The spark plasma sintering (SPS) technique was employed to prepare compacts from (i) gas-atomized and (ii) attritor-milled AE42 magnesium powder. Short attritor-milling was used mainly to disrupt the MgO shell covering the powder particles and, in turn, to enhance consolidation during sintering. Compacts prepared by SPS from the milled powder featured finer microstructures than compacts consolidated from gas-atomized powder (i.e., without milling), regardless of the sintering temperatures in the range of 400–550 °C. Furthermore, the grain growth associated with the increase in the sintering temperature in these samples was less pronounced than in the samples prepared from gas-atomized particles. Consequently, the mechanical properties were significantly enhanced in the material made of milled powder. Apart from grain refinement, the improvements in mechanical performance were attributed to the synergic effect of the irregular shape of the milled particles and better consolidation due to effectively disrupted MgO shells, thus suppressing the crack formation and propagation during loading. These results suggest that relatively short milling of magnesium alloy powder can be effectively used to achieve superior mechanical properties during consolidation by SPS even at relatively low temperatures.

## 1. Introduction

The fast development of new sintering and additive manufacturing methods continuously increases application possibilities of powder metallurgy. The spark plasma sintering (SPS) technique is nowadays considered to be one of the most advanced sintering techniques. This method, which involves high loads and well controllable high heating/cooling rates [1], is capable of retaining fine-grained microstructure because of short sintering times [2,3]. Besides, it can enhance densification in comparison with other techniques regardless of the thin oxide shell often present on the particles’ surface [4]. This technique is also capable of producing samples of complex shapes [5]. However, in magnesium alloys, the consolidation problems related to the presence of this shell and, consequently, to a limited diffusion are more severe than in other commonly processed metals. Oxides form immediately during the gas-atomization because some low concentration of oxygen is usually present in the chamber due to the safety reasons [6]. The high reactivity of magnesium results in formation of several times thicker oxide layer than e.g., in the case of aluminum [6]. Consequently, 3D net-like MgO phase distribution is usually present in consolidated samples along the former powder particles’ boundaries [7,8,9]. It was shown in several studies that such distribution of MgO has a negative effect on the ductility of bulk samples [7,8,10,11].

In the literature, there are reports showing possibilities to deal with the MgO shell present on the powders’ surface [11,12,13,14,15,16,17]. It is considered that it is crucial to remove or to fragment the oxide layer in order to increase diffusion during the sintering and to prevent continuity of the MgO structures in the consolidated samples. Three main approaches were elaborated in the related literature. The first one is the mechanical milling of powder in the protective atmosphere prior to the sintering [11,12,13]. This method is beneficial for fragmentation of the oxide shell; however, it is usually time-consuming. The second approach is focused on the tailoring of the SPS processing parameters. It was shown that a sufficient increase in the pressure [14] and sintering temperature might lead to better consolidation regardless of the oxide film [15]. There is also a report proposing the usage of high-frequency current during sintering, which causes the so-called skin effect on the powder surface and, consequently, the MgO tends to evaporate [16]. The third approach comprises the addition of alloying elements in which oxides feature higher thermodynamical stability [17]. 

This study focuses on the characterization of commercial magnesium alloy AE42 (4 wt.% of Al and 2 wt.% of rare earth (RE) mischmetal) prepared by sintering of (i) gas-atomized or (ii) milled powders. Unlike the typical approach, short mechanical milling prior to the sintering and utilization of high load during the sintering was employed in this work in order to enhance diffusion and improve material consolidation. Attritor-milling was used because it is capable of processing the powder faster in comparison to, e.g., ball-milling [18], and one hour of milling time was selected to fragment MgO while keeping the processing time short enough. The microstructure refinement due to milling is in this study considered only as a secondary effect.

The achieved results suggest that the short milling of the powder in the attritor under argon atmosphere can effectively facilitate better consolidation during the sintering. The microstructure of the sintered samples becomes finer and, more importantly, the mechanical properties such as ductility and ultimate strength are significantly improved because of the sufficient fragmentation of MgO shell prior to the sintering and change in the particles’ shape. The results indicate that this time-effective approach can be suitable for commercial production of bulk magnesium materials prepared by powder metallurgy.

## 2. Materials and Methods

Gas-atomized powder of the magnesium alloy AE42 (Mg-4 wt.%-Al-2 wt.% mischmetal, Clausthal University of Technology, Clausthal-Zellerfeld, Germany) was used as experimental material in this study. The powder was sieved, and the only fraction of 45–80 μm was used in this study. Subsequently, the powder was milled in Ar atmosphere using the Union Process 01-HD (London, UK) attritor equipped with a stainless-steel shaft and milling tank. The milling was performed at 300 rpm, using stainless-steel balls of 6.35 mm in diameter and the ball-to-powder ratio of 40:1 by weight. Only short milling for 1 h was used in this study as it is primarily focused on the disruption of the continuous oxide shell present on the gas-atomized powder particles. In order to reduce contamination after the milling, the powder was handled in a high purity argon-filled glove box at all times.

Sintering was performed in the SPS system—type 10-4, Thermal Technology LLC. (Santa Rosa, CA, USA). The holding time was selected to be 3 min, and the sintering temperatures were chosen to be 400, 450, 500, or 550 °C, based upon the previous investigations [9]. The maximum applied stress was 100 MPa. The consolidated samples are denoted as GA (gas-atomized) and M (milled) depending on the condition of the powder, e.g., GA-400C or M-400C. 

The microstructure of the powder particles and consolidated samples was investigated by scanning electron microscope (SEM) ZEISS Auriga compact equipped with EDAX EDX detector and EDAX EBSD camera (Berwyn, PA, USA). Samples for SEM were mechanically polished down to 1 µm diamond paste. Subsequently, samples for ESBD were ion-polished by Leica EM RES102. Otherwise, the samples were finally polished using the Struers Etosil suspension. The MgO content and crystallite size in the Mg matrix were investigated by X-ray diffraction (XRD) for both powder conditions. The measurement was performed by the Bruker D8 device (Billerica, MA, USA) at room temperature using the Cu_Kα_ radiation and the 1D LynxEye detector (Bruker). The phase composition and crystallite size were subsequently calculated by Rietveld refinement [19] using the TOPAS 5 software (Bruker).

Mechanical properties of the sintered samples were investigated by uniaxial compression deformation tests at room temperature. Samples having dimensions of 3 mm × 3 mm × 4.5 mm were cut from the consolidated samples with deformation axis perpendicular to the sintering loading direction, and, subsequently, the deformation tests were carried out using the INSTRON 5882 testing machine (Norwood, MD, USA) with the initial strain rate of 10^−3^ s^−1^. Four samples were tested for each investigated condition. 

## 3. Results

### 3.1. Effect of RT Milling on the Powder

**Figure 1:**
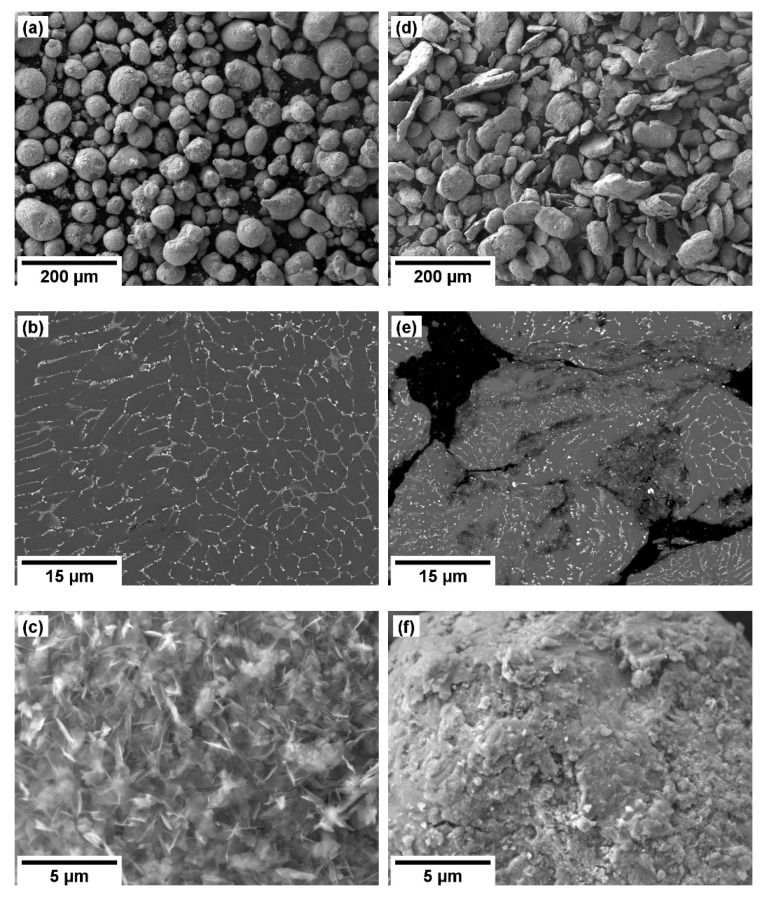
SEM micrographs of gas-atomized powder: (**a**) overview, (**b**) detail of microstructure, and (**c**) detail of the surface. SEM micrographs of attritor-milled powder: (**d**) overview, (**e**) detail of microstructure, and (**f**) detail of the surface.

The as received, gas-atomized, powder particles exhibited a typical round/ellipsoidal shape and contained very fine eutectic lamellar phases, see Figure 1a,b.

**Figure 2:**
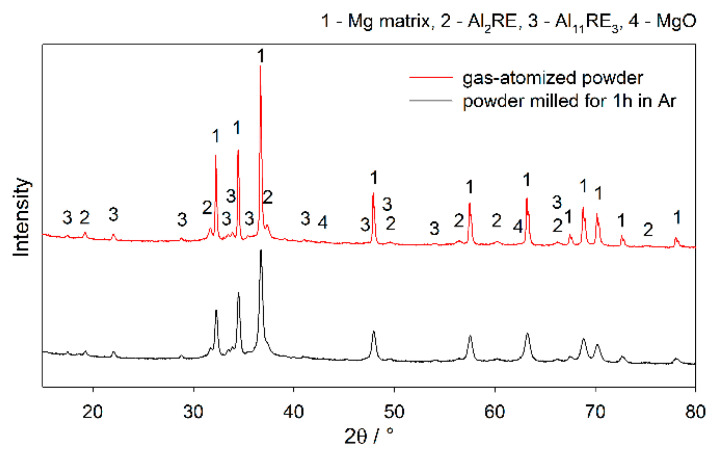
XRD diffraction pattern of the gas-atomized and attritor-milled powder.

These secondary phases were identified as a mixture of Al_11_RE_3_ and Al_2_RE phases by the XRD diffraction measurement, see Figure 2.

Severe plastic deformation introduced to the powder particles by the attritor milling at room temperature had a significant effect on their morphology and their inner structure. The milling imposed substantial straining on the particles and fragmentation of the eutectic lamellae, as well as deformation and crumbling of the whole powder particles took place. Consequently, the milled powder particles exhibited an irregular shape, as shown in Figure 1d. The crystallite size of Mg matrix calculated from the XDR pattern decreased from ~375 nm to ~40 nm after the milling. Note that crystallite size corresponds to the coherently diffracting zone and not to grain size. Interestingly, the average particle size did not change despite the milling. Closer examination revealed that the individual particles were repeatedly crushed and cold-welded together, see Figure 1e.

Furthermore, a significant additional effect caused by the milling was observed. Figure 1c shows that the as-received particles were covered with a continuous layer of the oxide film. As expected, the milling caused disruption and fragmentation of this film, see Figure 1f. The XRD measurement performed on both the gas-atomized and milled powders showed that the MgO amount increased from 0.8(2) to 1.4(2) wt.%. Note that such a minor increase may be attributed to the oxidation during the manipulation with powder prior to the XRD measurement, because the XRD device was not equipped with the vacuum chamber. However, all the manipulation before the sintering was performed in the glovebox filled with high-purity argon. Therefore, excessive oxidation was not expected. 

### 3.2. Microstructure of Sintered Samples

The microstructure of the samples consolidated from the milled powder exhibited two significant differences in comparison to the samples sintered from the gas-atomized one, regardless of the sintering temperature. All samples sintered from the milled powder had a significantly more refined grain structure. In addition, the substantial change in the shape of the powder particles because of the milling resulted in a distinctively different microstructure with respect to the distribution of MgO. On the other hand, the sintering of powder in both conditions resulted in comparably good densification. Residual porosity estimated by SEM was in all investigated samples 1% or less. 

**Figure 3:**
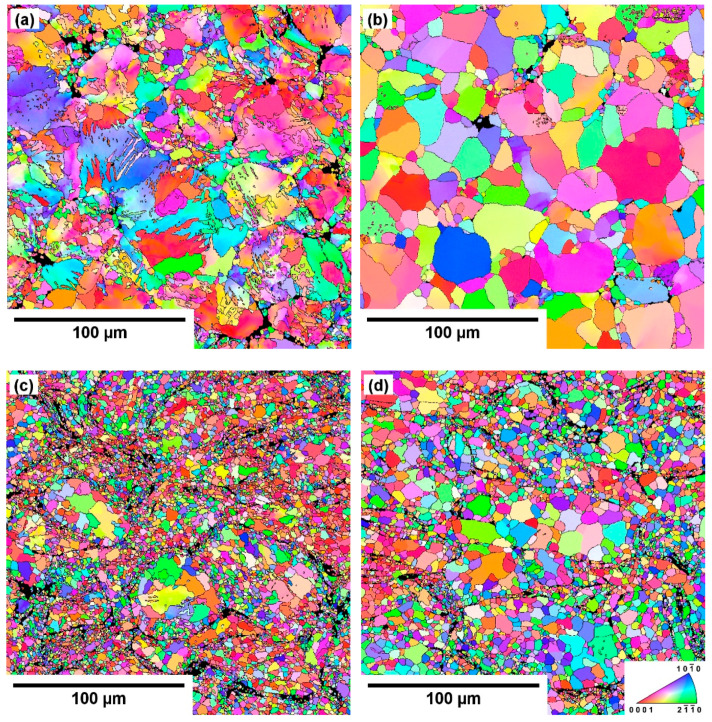
EBSD orientation maps of (**a**) GA-400C, (**b**) GA-550C, (**c**) M-400C and (**d**) M-550C samples. IPF colored along the sintering load direction.

**Figure 4:**
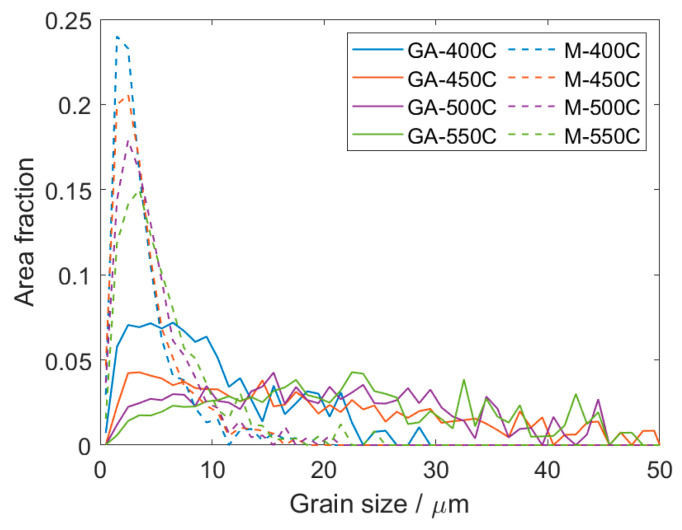
Grain size distribution calculated from EBSD data for all investigated samples.

The effect of milling on the grain structure is well visible in the EBSD orientation maps shown in Figure 3, as well as on the grain size distribution plot shown in Figure 4.

The significant difference between the samples sintered from the milled and gas-atomized powder was in the average grain size and uniformity of the microstructure. The average grain size measured in the GA-400C sample was ~10 µm, while in the case of the M-400C sample, it was only ~4 µm. However, as disclosed in Figure 4, the grain size distribution of the GA-400C sample was much broader. The prominent peak corresponds to the grain size of ~5 µm and represents grains that are not only present along the former particles’ boundaries but also in large grain interiors, see Figure 3a. The distribution also exhibits plateau corresponding to grains with a diameter around ~18 µm, which were observed almost in all former powder particles, as disclosed in Figure 3a. Note that the average grain size was calculated as a weighted average utilizing area fraction of each grain size as a weight. The resulting average grain size then reflects the average grain boundary density in the material. In the case of the M-400C sample, the microstructure was more uniform regardless of the fact that larger grains were predominantly observed in the former powder particle interiors also in this sample (Figure 3c). However, the grain size distribution is rather sharp and reaches its maximum at ~2 µm.

An increase in the sintering temperature resulted in the grain growth in both types of samples. However, the initial powder condition still had a dominant effect. In the case of the samples sintered from gas-atomized powder, the grain growth caused a gradual broadening of the grain size distribution up to the sintering temperature of 550 °C, as shown in Figure 4. The bimodal grain size distribution observed in the GA-400C sample completely disappeared in the case of the GA-550C sample, and the grain structure was formed by grains with an average size of ~22 µm. The grain size distribution was very broad and comprised grains up to 50 µm in diameter, see Figure 4. On the other hand, grain growth in the samples sintered from the milled powder was not so pronounced with an increase in the sintering temperature. The average grain size of the M-550C sample increased only to ~6 µm, but the grain size distribution became partially bimodal, as depicted in Figure 3d and Figure 4. The occurrence of grains with size above 15 µm corresponds to much faster grain growth in some former powder particles, especially in their inner part, see Figure 3d. The average grain size values calculated for all studied samples are shown in Table 1.

The EBSD investigation also revealed that both types of samples sintered at 400 °C, but especially the GA-400C sample, exhibited a relatively high residual strain observed as a variety of color shades within individual grains. A qualitatively lower amount of internal strain observed in the M-400C sample can be associated with a much higher degree of recrystallization resulting from higher deformation of the powder particles before the sintering. Nevertheless, the increase in the sintering temperature caused a significant decrease in the residual strain, and the microstructure of both types of samples sintered at 550 °C appears to be practically strain-free. 

**Figure 5:**
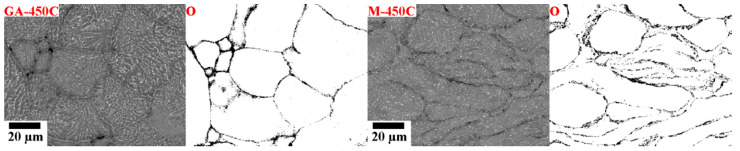
SEM micrographs of GA-450C and M-450C samples and the corresponding oxygen EDX.

As mentioned above, the second significant effect of the milling was related to the MgO distribution in the sintered samples. Continuous MgO layer present on the surface of the round/ellipsoidal gas-atomized powder particles resulted in the formation of 3D net-like MgO structures. However, because of the particles’ shape, the majority of the interacting angles in this MgO structure became obtuse with a high angle, as depicted in Figure 5.

On the other hand, the milling significantly changed the particles’ shape and the MgO layer was considerably disrupted. The majority of the particles exhibited irregular shape, and many of them were strongly fragmented and repeatedly cold-welded during milling. Consequently, the MgO distribution in the material became more complex, see Figure 5. Moreover, the MgO layer was not as continuous as in the samples sintered from the gas-atomized powder. Therefore, it is assumed that the diffusion through the particles’ surface layer was more intensive, and the consolidation of the milled powder was more effective than in the case of its gas-atomized counterpart, irrespective of the sintering temperature.

### 3.3. Mechanical Strength of the Sintered Samples

**Figure 6:**
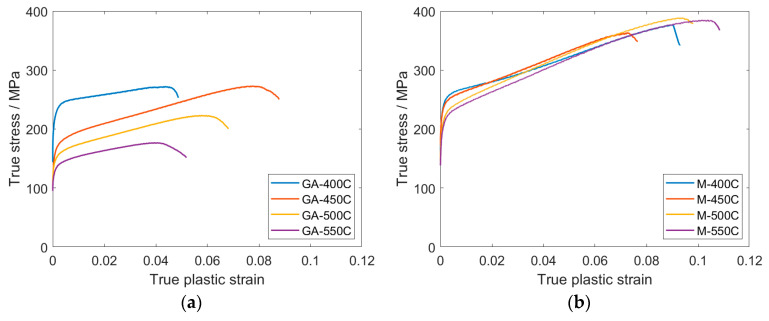
Representative true stress—true strain curves for (**a**) gas-atomized and (**b**) milled samples measured in compression.

The effect of a relatively short (1 h) attritor-milling in Ar atmosphere on the mechanical strength of the sintered material was substantial. Figure 6 shows true stress—true strain curves for all investigated samples deformed in compression and the calculated values of yield compression strength (YCS), ultimate compression strength (UCS), and strain corresponding to UCS (ε_max_) are shown in Table 1.

It is clear that the short milling caused a significant increase in both the YCS and UCS values, considering the same sintering temperature. The YCS values of both types of samples, with respect to the powder condition, continuously decreased with the increasing sintering temperature in the range of 400–550 °C. However, this decrease was more pronounced in the gas-atomized samples in which YCS decreased from ~236 MPa to ~137 MPa. On the other hand, YCS of milled samples decreased from ~250 MPa to ~210 MPa. Considerably different evolution was observed for the UCS values. A significant decrease in the UCS values with increasing sintering temperature was observed in gas-atomized samples. In contrast, in the case of the samples sintered from the milled powder, the UCS values were comparable in all samples. Values of ε_max_ were systematically lower in the gas-atomized samples except for the GA-450C sample, which was comparable with the M-450C sample. It should be noted that the deformation curves were very similar within the same type of material, leading to relatively low uncertainties in Table 1. In this regard, microstructure in the sintered samples can be considered as macroscopically consistent and uniform.

## 4. Discussion

The short attritor-milling of the powder imposed three primary effects on the microstructure of the bulk samples. Firstly, high strain introduced to the powder particles caused the formation of much finer microstructure after the sintering, regardless of the sintering temperature. Secondly, the disruption of the MgO layer caused better consolidation during the sintering by promoting diffusion between individual powder particles. And, finally, changes in the shape of the powder particles had a significant impact on the MgO distribution in the sintered samples.

The effect of mechanical milling utilized prior to the sintering of various magnesium alloys was already investigated using different milling techniques, milling media, and temperatures [12,13,20,21,22,23]. Usually, the primary interest was to achieve (ultra)fine-grained microstructure in the sintered samples. The most refined microstructure in the sintered magnesium alloy sample, having the average grains size of 45 nm, was achieved after cryo-milling in liquid nitrogen and subsequent sintering at 400 °C. However, much larger grains (~10 μm) were observed in the corresponding micrographs, and therefore the authors’ claim is highly suspicious [22]. The reports focusing on the room temperature milling showed that long milling times are necessary to achieve saturation in the powders’ microstructure refinement. The grain size of ~80 nm (determined by transmission electron microscope) was achieved after 20 h of high-energy ball-milling [13], and the crystallite size of 40 nm (determined by XRD), which is comparable with the results shown in this study, were achieved after 10 h of shaker-milling [12] and after 5 h of planetary ball-milling [24]. Therefore, a relatively high effectivity of the attritor-milling in the microstructure refinement of magnesium alloys is in good agreement with our initial assumption. Nevertheless, the microstructure investigation in the sintered sample showed that the refinement is not uniform, and some former powder particles exhibit relatively large grains in their interiors, see Figure 3c. Therefore, the milling time needs to be increased if more uniform microstructure is needed. However, as mentioned in the Introduction, this study was primarily focused on the disruption of the MgO shell while keeping the processing time very short. In this regard, it was shown that the MgO layer present on the powders’ surface was successfully disrupted (Figure 1f) and the powder particles were heavily strained and fragmented (Figure 1e). The observed change in the shape is consistent with the previous studies in which milling was performed at room or cryogenic temperature, see e.g., [12,22]. 

The subsequent sintering of the samples prepared from both powder types resulted in the formation of significantly different microstructures. Grain refinement observed in the samples sintered from the milled powder was not unique in comparison with the related literature [12,13,20,21,22,23]. On the other hand, as mentioned above, higher milling times are typically utilized. As expected, besides the pre-milling effect, also the impact of the sintering temperature was observed, albeit it was relatively less pronounced. The grain growth in the samples sintered from the gas-atomized powder was observed only when the sintering temperature was increased from 400 °C to 450 °C. Subsequent temperature increase up to 550 °C did not result in further statistically significant grain growth. These results are in good agreement with our previous study, which was focused solely on the samples sintered from the gas-atomized AE42 powder in the temperature range of 450–550 °C, using different SPS device and lower external load [9]. The results of this study showed that the highly limited grain growth in this temperature range could be associated with the initial microstructure of the powder particles [9]. Significantly smaller average grain size in the GA-400C sample can be related to strain localization and twinning during SPS, see Figure 3a. Exposure to the lowest temperature of 400 °C resulted in only partial recrystallization. It is worth recalling that variations in color within individual grains representing the orientation of each pixel in the EBSD orientation maps indicated that there was a lot of residual strain present in the GA-400C sample. Small newly formed grains corresponded to the occurrence of ~5 μm peak in Figure 4. An increase in the sintering temperature above 450 °C resulted in more intensive grain growth, and the small grains became more likely to be absorbed by their larger neighbors (see the decreasing fraction of grains smaller than 10 μm in Figure 4 with increasing sintering temperature) [25]. On the other hand, the microstructure of milled powder particles was heavily strained before the sintering. Therefore, the recrystallization, which was initiated during sintering, was more uniform, and even at high temperatures, a relatively high fraction of small grains persisted, as shown in Figure 4. As mentioned above, the occurrence of much larger grains in the former powder particles’ interiors corresponds to unsaturated straining introduced by the milling. In these areas, a lower amount of new grains formed and, therefore, they became larger [26]. The overall decrease in the residual strain with increasing sintering temperature is a common feature observed before, see, e.g., [27].

The average grain size in the consolidated samples significantly affected the yield compression strength, especially if samples sintered from different powder types are compared. However, it cannot solely explain the massive drop in the yield compression strength with increasing sintering temperature in the samples sintered from the gas-atomized powder, especially in the temperature range 450–550 °C, where the average grain size was comparable within the experimental uncertainty (Table 1). The existence of such a drop was elucidated in the previous study to be a result of globularization of fine Al_11_RE_3_ lamellae observed in the microstructure of the gas-atomized powder and a consequent decrease in the density of these secondary phase particles [9]. On the other hand, this effect was not so significant in the samples sintered from the milled powder. A much higher density of grain boundaries in these samples could effectively hinder dislocation movement, regardless of the changes in size, shape, and density of the secondary phase particles, which were otherwise comparable to the samples sintered from gas-atomized powder (not shown here). Therefore, this explains why the deterioration of YCS was not so pronounced in the samples made of milled powder. 

The YCS values achieved in the samples sintered from the milled powder are difficult to be directly compared with other studies as there are only a few works presenting compression yield strength of the samples sintered from the short-milled magnesium powder. As mentioned above, these studies were primarily focused on the microstructure refinement and, therefore, the achieved YCS values were usually much higher. For example, in cryo-milled AZ80, YCS of 442 MPa was achieved [20], and in cryo-milled AZ31, YCS of 400 MPa was reported [22], but in both studies, the average grain size was at least one order of magnitude lower. On the other hand, sintering of a heavily milled pure Mg resulted in coarser microstructure and, consequently, comparable grain size and mechanical strength were achieved—SPS of the cryo-milled pure Mg resulted in YCS of 179 MPa [28] and in the case of the planetary ball-milled pure Mg at room temperature, YCS was 236 MPa [24]. Nevertheless, comparable grain size (~3 μm) was also achieved after the sintering of the gas-atomized AZ91 alloy, and the measured YCS was 230 MPa [7]. Therefore, it can be concluded that the results achieved in this study are consistent with the literature. 

The other, even more important benefit stemming from the short milling times of the gas-atomized powder is associated with the high UCS attained in all samples sintered from the milled powder. UCS of ~380 MPa was achieved regardless of the sintering temperature. As mentioned in the Introduction, poor deformability of samples sintered from the gas-atomized magnesium has been reported in several studies and was directly associated with the MgO layer present along the former powder boundaries [7,8,10,11]. Concerning the powder metallurgy technique, a decrease in the ductility is often associated with the residual porosity and coalescence of pores and microcracks during the loading [29]. At the same time, the formation of 3D net-like structure of the brittle MgO phase represents an additional weak point for crack propagation. A similar problem was recently revealed also in the case of low-alloyed steel prepared by powder metallurgy, where crack tended to propagate along with the Ni-rich ferrite present as an envelope around more ductile austenite particles [30]. As shown above, mechanical milling significantly disrupted the MgO shell forming the powder particles’ surface, leading to enhanced diffusion and more effective consolidation during the sintering. The MgO distribution became more random and intermittent. Therefore, the growth of microcracks, which form during straining, was effectively obstructed by the presence of well-consolidated areas. In addition, intergranular crack propagation was more effectively hindered by the fine-grained microstructure [31], and significant change in the powder particles’ shape resulted in a more random distribution of MgO. Consequently, the samples sintered from the milled powder sustained a much higher load before failure. 

## 5. Conclusions

A short attritor-milling of the gas-atomized AE42 magnesium powder was performed in order to fragment MgO shell usually present on the powder particles’ surface and, in turn, to improve consolidation during sintering. Subsequently, two sets of samples, i.e., from the gas-atomized and milled powders, were sintered using SPS with sintering temperature in the range of 400–550 °C and 3 min holding time. The study was primarily focused on the effect of short milling on the microstructure and mechanical properties of the sintered samples, and the main conclusions are as follows:

- The attritor-milling performed for one hour, at room temperature and under Ar atmosphere, led to significant deformation of the individual powder particles. Consequently, the particles’ shape changed from sphere/ellipsoid-like to irregular, the crystallite size decreased from ~375 nm to ~40 nm, and the MgO layer present on the particles’ surface was significantly disrupted.
- Consolidation performed by SPS led to the formation of microstructure, which was significantly dependent primarily on the powder condition, but also the sintering temperature. Much finer grain structure was observed in the samples sintered from the milled powder regardless of the sintering temperature. Moreover, grain growth associated with an increase in the sintering temperature in these samples was less pronounced compared to ones consolidated from the gas-atomized powder.
- The yield compression strength, deformability, and also the ultimate compression strength were significantly affected by the short milling of the gas-atomized powder. In the samples sintered from the milled powder, an increase in the yield strength was primarily associated with a much more refined grain structure. A significant increase in the ultimate compression strength and deformability was attributed to the synergic effect of a more refined grain structure, better consolidation due to fragmented MgO shell on powders, and irregular shape of the milled powder particles. It can be assumed that the combination of all these attributes effectively hinder crack propagation during loading.
- It was shown that one hour of attritor-milling can significantly improve the consolidation of magnesium alloys by SPS, resulting in substantially enhanced mechanical properties. In addition, it was revealed that even relatively short milling of initial powder could result in excellent mechanical properties after the sintering at the temperature as low as 400 °C.

## Figures and Tables

**Table 1:** Yield compression strength (YCS), ultimate compression strength (UCS), and strain corresponding to UCS (ε_max_) calculated for all investigated samples.

Powder Condition	Gas-Atomized				Milled			
Sintering Temperature	400 °C	450 °C	500 °C	550 °C	400 °C	450 °C	500 °C	550 °C
**GS/μm**	10(1)	20(2)	21(1)	22(2)	4.0(2)	4.2(2)	4.7(3)	5.7(3)
**YCS/MPa**	236(7)	179(5)	154(5)	137(4)	250(9)	242(5)	233(14)	210(12)
**UCS/MPa**	273(8)	277(8)	219(11)	171(6)	388(15)	365(8)	386(9)	383(9)
**ε_max_**	0.04(1)	0.08(1)	0.06(1)	0.04(1)	0.09(1)	0.07(2)	0.08(2)	0.10(1)
